# Effect of Transmural Differences in Excitation-Contraction Delay and Contraction Velocity on Left Ventricle Isovolumic Contraction: A Simulation Study

**DOI:** 10.1155/2018/4798512

**Published:** 2018-05-10

**Authors:** J. Vaverka, J. Burša, J. Šumbera, M. Pásek

**Affiliations:** ^1^Institute of Solid Mechanics, Mechatronics and Biomechanics, Faculty of Mechanical Engineering, University of Technology, Brno, Czech Republic; ^2^Department of Cardiovascular Diseases, Faculty of Medicine, Masaryk University, Brno, Czech Republic; ^3^Department of Physiology, Faculty of Medicine, Masaryk University, Brno, Czech Republic; ^4^Institute of Thermomechanics, Czech Academy of Science, Prague, Czech Republic

## Abstract

Recent studies have shown that left ventricle (LV) exhibits considerable transmural differences in active mechanical properties induced by transmural differences in electrical activity, excitation-contraction coupling, and contractile properties of individual myocytes. It was shown that the time between electrical and mechanical activation of myocytes (electromechanical delay: EMD) decreases from subendocardium to subepicardium and, on the contrary, the myocyte shortening velocity (MSV) increases in the same direction. To investigate the physiological importance of this inhomogeneity, we developed a new finite element model of LV incorporating the observed transmural gradients in EMD and MSV. Comparative simulations with the model showed that when EMD or MSV or both were set constant across the LV wall, the LV contractility during isovolumic contraction (IVC) decreased significantly ((*dp*/*dt*)_max⁡ _ was reduced by 2 to 38% and IVC was prolonged by 18 to 73%). This was accompanied by an increase of transmural differences in wall stress. These results suggest that the transmural differences in EMD and MSV play an important role in physiological contractility of LV by synchronising the contraction of individual layers of ventricular wall during the systole. Reduction or enhancement of these differences may therefore impair the function of LV and contribute to heart failure.

## 1. Introduction

Myocardial infarction and heart failure belong to leading causes of death in the Western world. Both the clinical practice and laboratory research have shown that the mechanical performance of left ventricle (LV) is one of the most important factors that affect the pump function of the heart. Thus, understanding the processes underlying the physiological and pathophysiological function of LV is unavoidable for efficient treatment of reduced LV contractility. Computational modelling has been proposed and actively pursued as a tool for accelerating research in cardiac biomechanics. Up to now, many attempts were done to describe mechanical properties of the heart and to simulate their impact on cardiovascular hemodynamics under health and disease (for latest reviews see [1–3]).

One of the favourite approaches enabling simulation of the effect of electromechanical properties of cardiac muscle on development of tension in LV wall and intraventricular pressure during heart contraction is based on three-dimensional finite element (FE) models (e.g., [4–11]). To approach the real conditions of LV, studies published to date proposed the models with respect to (i) a real (asymmetric) geometry of human LV [5–8, 12]; (ii) electromechanical interaction with the right ventricle (RV) [6, 10]; (iii) transmural variation in fibre orientation [4–8, 10–12]; (iv) sequential activation of different heart regions [10, 11]; (v) viscoelastic behaviour of the myocardial tissue [4, 13].

In 2004, Cordeiro et al. [17] showed that the time period between local electrical depolarization and onset of local shortening of myocytes (electromechanical delay: EMD) and the myocyte shortening velocity (MSV) varies transmurally in the ventricular myocardium. This reflects intrinsic differences in characteristics of membrane transport systems and excitation-contraction coupling in the myocytes from different layers of the ventricle [17, 18]. Later, Campbell et al. [19] embedded a cellular excitation-contraction coupling model with region-dependent parameters in a simple FE model of LV to study the effects of transmural electromechanical heterogeneity on local myocardial mechanics and global hemodynamics. Their study showed that a random assignment of parameters related to subendo-, midmyo-, and subepicardial cells to the LV model elements (i.e., a transmurally homogenous arrangement of the myocyte subtypes) affected significantly the transmural patterns of fibre and cross-fibre strains during early systole, but not in its later phases. Consequently, calculated parameters of the LV hemodynamic function (peak LV pressure, the maximum rate of its rise, and stroke volume) were almost identical to those in the control model in which the myocyte parameters were specified per elements according to myocardial region (transmurally heterogeneous arrangement of myocyte subtypes). However, the physiological importance of transmural differences in EMD and MSV during the systolic phases was not studied. Therefore, the aim of our work was to incorporate these wall depth dependent differences in the mechanical activity of cardiomyocytes into a newly developed LV model and to explore their role in the physiological function of LV during isovolumic contraction (IVC).

## 2. Methods

### 2.1. Geometry and FE Discretization

**Figure 1:**
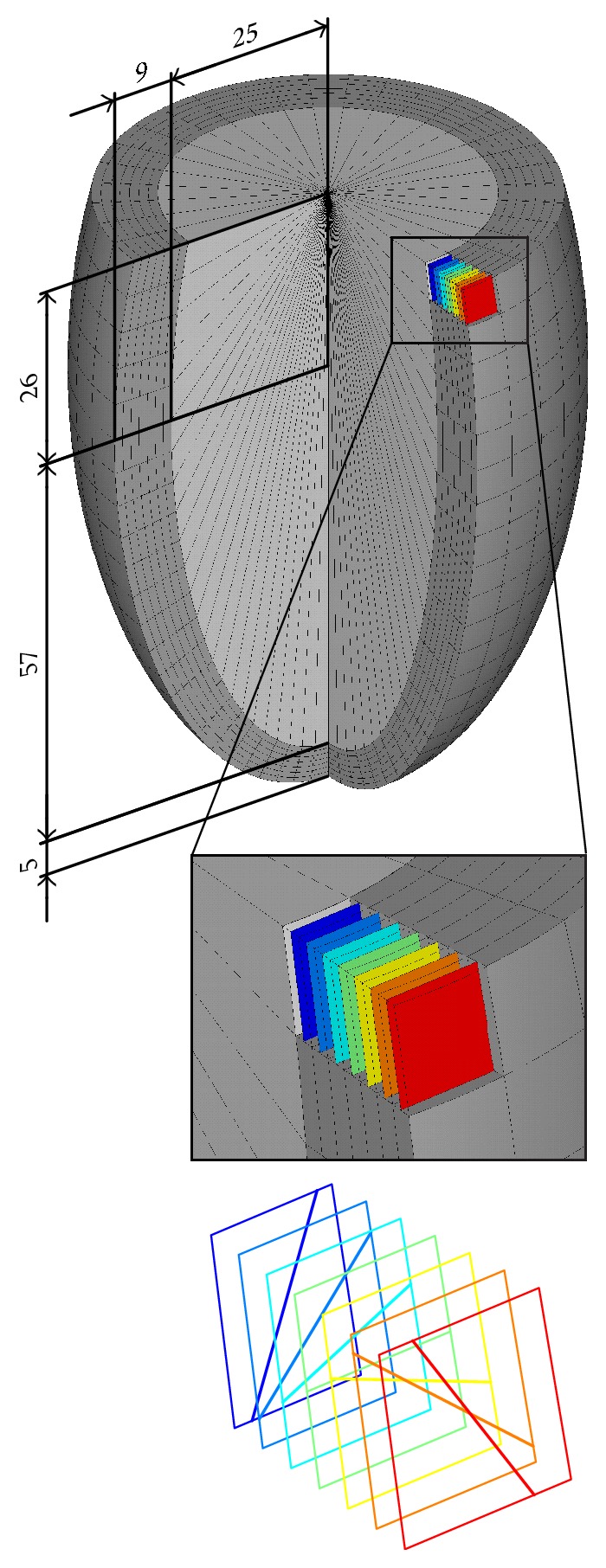
Geometry of the finite element model of left ventricle. The model consists of 7 layers (see details in the middle part), each of them containing differently oriented elements representing muscle fibres (bottom part).

To simulate the ventricular contraction, a three-dimensional FE model was built using commercial FE software ANSYS®. Since the anatomical realism is not crucial for the problem addressed in this study, the model was based on a simplified geometry. The inner cavity and the outer surface of the ventricle are approximated by ellipsoids with the same axes of symmetry, truncated by a plane perpendicular to their major axis mimicking the LV basal plane. The dimensions were set according to literature (see Figure 1) to define the LV end-diastolic volume of 122 ml.

The myocardial wall was discretized with 6048 quadratic hexahedral solid elements arranged in 7 layers. To mimic the active contraction of the myocytes, an additional reinforcing element in the form of a membrane parallel to both LV surfaces was embedded within each solid element. This membrane exhibits a unidirectional stiffness only to model the fibres aligned in a given direction. The angle of fibres against the circumferential direction (helix angle) varies linearly across the 7 layers from +60° in the inner (subendocardial) layer to −60° in the outer (subepicardial) layer [20, 21]. The thickness of the reinforcing membrane was set to occupy 80% of volume of the respective solid element which reflects the volume fraction of myocytes in ventricular myocardium [22]. The blood volume inside the cavity was modelled as an incompressible liquid using special hydrostatic fluid elements capable of modelling fluids fully enclosed by solids. The mesh density (Figure 1) was proven to be sufficient for the analysis.

### 2.2. Passive Properties of Myocardium

To describe the passive mechanical behaviour of myocardium, an incompressible transversely isotropic hyperelastic model was used. The preferred direction of the model representing the direction of muscle fibres was set for each solid element individually following the same rule as mentioned above for the reinforcing elements. The model was defined by a polynomial strain energy density function having the form

(1) $$\begin{array}{c} W=\overset{3}{\underset{i=1}{\sum }}a_{i}{\left(\;I_{1}-3\;\right)}^{i}+\sum_{j=2,4}b_{j}{\left(\;I_{4}-1\;\right)}^{j}, \end{array}$$

where *a*_*i*_ and *b*_*j*_ are stress-like material parameters, *I*_1_ is the first invariant of right Cauchy-Green deformation tensor describing the isotropic part of deformation, and *I*_4_ is (pseudo) invariant of the right Cauchy-Green deformation tensor representing squared stretches in the fibre directions.

The material parameters were obtained as the best fit of different biaxial tests of myocardium published by Sommer et al. [23]; using our inhouse software Hyperfit (http://www.hyperfit.wz.cz) the following values were obtained: *a*_1_ = 0,347 kPa, *a*_2_ = 13,438 kPa, *a*_3_ = 48,846 kPa, *b*_1_ = 0,436 kPa, and *b*_2_ = 27,692 kPa. Since the passive response of myocardium is rate-dependent (effect of viscoelasticity), we utilized the results from equibiaxial tests performed at different testing speeds as well as from relaxation tests [23] and estimated that under strain rates typical for normally beating heart the myocardial stiffness is approximately 6 times higher compared with the normal testing speed. Therefore the above constants were multiplied by a factor of 6 in our model.

### 2.3. Active Fibre Contraction

As mentioned above, active contraction was incorporated into the model through reinforcing elements oriented in the local directions of myocytes in LV myocardium. To simulate heterogeneous time-dependent unidirectional contraction of myocytes, a simple approach based on prescribing time-dependent (negative) thermal strain in the fibre direction was used. A coefficient of thermal expansion was prescribed and the time course of contraction was governed by a gradual decrease in fictitious temperature (having no relation to real temperature or thermal properties of myocardium). The unidirectional stress-strain relation for activated myocytes was estimated from the observed linear relation between force and length in fully contracted isolated cardiomyocytes. This relationship was independent of the mode of contraction and initial sarcomere length [24] and conforms to the formula

(2) $$\begin{array}{c} \sigma =E_{0}\cdot \exp\left(\;\varepsilon \;\right)\cdot \left(\;\exp\left(\;\varepsilon \;\right)-1\;\right), \end{array}$$

where *σ*, *ε*, and *E*_0_ = 200 kPa are Cauchy stress, logarithmic strain, and initial Young's modulus, respectively. Due to limitations of ANSYS program, the above relation was incorporated into the model using a concept of multilinear elasticity with zero compression stiffness.

### 2.4. Boundary Conditions

In each simulation, the onset of contraction was calculated for each contractile element individually taking into account the time course of electrical activation of LV myocardium and the transmural differences in EMD. The electrical activation was assumed to start simultaneously on the whole endocardial surface and to spread towards the epicardial surface with the velocity of 47 cm/s [25, 26]. The time of electrical activation was calculated as the ratio of the shortest distance of centroid of a particular element from the cavity surface and the conduction velocity.

As exact measurements of EMD and MSV across the wall of human LV are missing we implemented these features in our model on the basis of measurements from dog hearts published by Cordeiro et al. [17]. They show a decrease of EMD in LV wall of 9 mm thickness from 47 ms (subendocardium) to 28 ms (subepicardium); thus we used a transmural EMD gradient of 2.1 ms/mm to simulate IVC under control conditions. In conjunction with the conduction velocity of 47 cm/s, this resulted in a simultaneous contraction of all contractile elements because the transmural depolarization gradient was completely counterbalanced by the EMD gradient.

The time course of contraction (controlled by temperature decrease; see Section 2.3) was defined on the basis of literature: a sequence of temperature values was prescribed to each contractile element to reproduce the experimental shortening traces of unloaded cells. As the initial part of shortening trace including IVC is typically fairly linear [27–29], we assumed a linear shortening of myocytes. Consistent with the experimental traces from Cordeiro et al. [17, 30], the MSV in subepicardial layer was set two times higher than in subendocardial layer with linear decrease between them. The actual values were determined so that the intraventricular pressure increased from its end-diastolic value (1,3 kPa) to diastolic aortic pressure (10,7 kPa) in 60 ms (normal value of IVC time [31]). This normally contracting ventricle is denoted as the control model below.

The nonzero end-diastolic pressure causes the real ventricular wall to not be unloaded at the beginning of contraction. Thus the FE mesh in Figure 1 cannot represent the unloaded LV configuration and application of end-diastolic pressure on this geometry would lead to incorrect shape and volume of LV at the beginning of IVC. Therefore we employed an iterative algorithm based on the approach proposed by Bols et al. [32] to compute the unloaded (zero-pressure) geometry. As a result of the iterative process, the zero-pressure geometry was obtained with intraventricular volume of 105 ml. Upon the application of end-diastolic pressure to the hydrostatic fluid elements, the original ellipsoidal shape was restored and simulation of IVC begins. In all these simulations zero displacements were imposed on all nodes at the basal plane to model the firm attachment of LV myocardium to the stiff collagenous skeleton. As the end-diastolic pressure is balanced only by passive wall stresses with myocytes being relaxed, the contractile elements were inactivated during the initial “filling” phase until the contraction begins.

In addition to the control model, three artificial sets of boundary conditions were simulated to explain the role of heterogeneous EMD and MSV. First, we combined the layer-dependent MSV with EMD being uniform in the whole model. Thus the mechanical activation was asynchronous having the same pattern as the excitation front. Secondly, the subendocardial MSV was prescribed to all layers while the EMD time were kept the same as in the control simulation. Finally, both EMD and MSV were considered constant in the LV myocardium.

## 3. Results

### 3.1. Intraventricular Pressure and Wall Stress in Control Model

**Figure 2:**
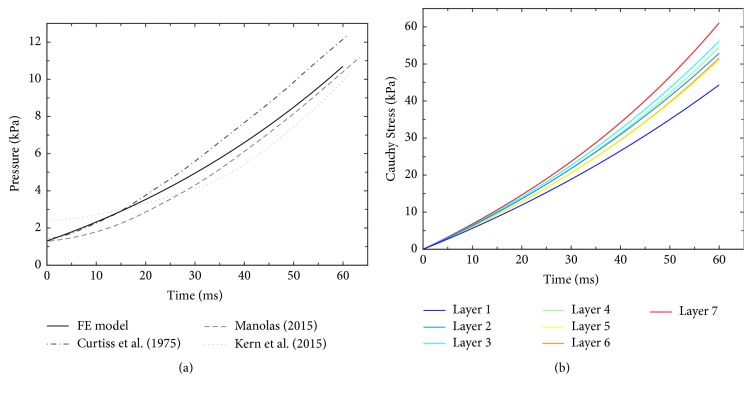
Time course of intraventricular pressure rise during IVC and the underlying increase of tension in LV wall under control conditions. (a) Simulated rise of intraventricular pressure and its comparison with digitalized experimental records published by Curtiss et al. [14], Manolas [15], and Kern et al. [16]. Note that the duration of IVK (∼60 ms) and *dp*/*dt*_max⁡_ (1780 mm Hg/s) agrees with clinically measured values (61 ms, 63 ms, 61 ms, and 1790 mm Hg/s, 1730 mm Hg/s, and 2130 mm Hg/s, resp.). (b) Simulated rise of (active) stress along the direction of myocytes in the individual layers. The colours of individual lines (from blue to red) denote the layers of LV wall (from endocardium to epicardium - see Figure 1).

Behaviour of the control model is illustrated in Figure 2(a) showing a time course of intraventricular pressure rise from its end-diastolic value of 1.3 kPa (10 mm Hg) up to 10.7 kPa (80 mm Hg) within 60 ms. This is fully consistent with the physiological rise of intraventricular pressure measured in human LV during the preejection period (see digitalized experimental traces in Figure 2(a)). Regarding the maximum rate of intraventricular pressure rise (*dp*/*dt*)_max⁡_, the model generates a value of 237.3 kPa/s (1780 mm Hg/s) that falls into the range of physiological values reported in the literature [33] and is attained in the end of IVC, just like in normal human heart [34]. These values show that the control model is capable of reliably simulating the physiological changes of intraventricular pressure during IVC in a real heart.  Figure 2(b) shows a related time course of stress (tension) development in individual layers. It is apparent that during IVC the tension in the subepicardial layer is higher than in subendocardial layer. However, its transmural change is not monotonous and under control conditions is reduced in the inner parts of the LV wall (see Figure 4).

### 3.2. Effect of Transmural Differences in Electromechanical Delay and Myocyte Shortening Velocity on Intraventricular Pressure Rise during Isovolumic Contraction

**Figure 3:**
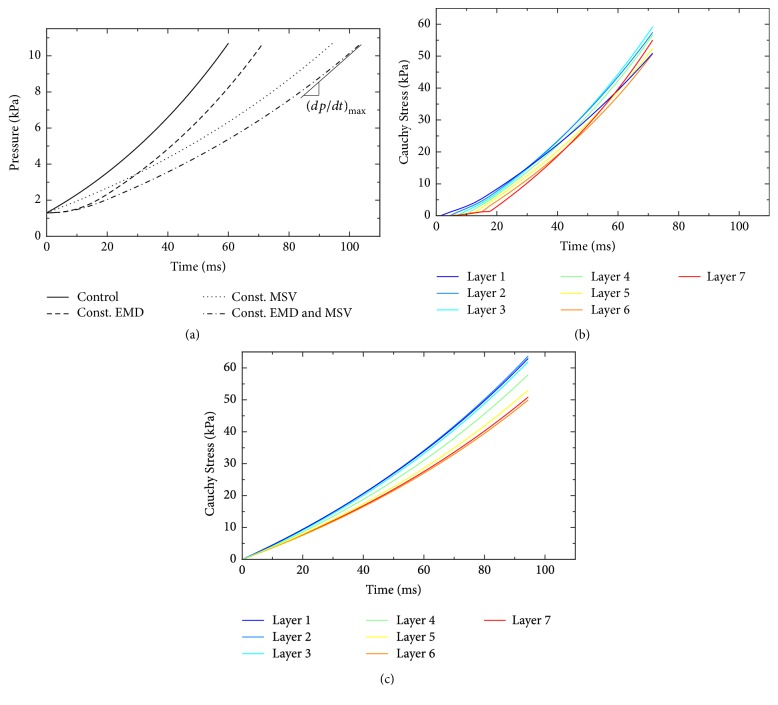
Effect of transmural differences in the electromechanical delay (EMD) and in the myocyte shortening velocity (MSV) on the rate of ventricular pressure rise (*dp*/*dt*), duration of isovolumic contraction (IVC) and the underlying tension in LV wall. (a) Simulated increase of intraventricular pressure under control conditions and when either EMD, or MSV, or both were set constant in all ventricular layers. (b) Simulated rise of tension in individual layers when EMD was set constant across the LV wall. (c) Simulated rise of tension in individual layers when MSV was set constant across the LV wall. Colours of the individual lines in (b) and (c) denote the layers of LV wall from subendocardium (blue) to subepicardium (red).

The transmural differences in EMD and MSV belong to the important characteristics of LV closely related to its function. To explore the physiological importance of the transmural decrease of EMD (see Section 2.4) in the function of LV during IVC, we compared the simulated time course of intraventricular pressure rise in the control model with that in which EMD was set constant (47 ms) in all ventricular layers. The results presented in Figure 3(a) (full and dashed lines) clearly show that the transmural homogeneity of EMD leads to a prolongation of IVC from 60 to 71 ms. However, (*dp*/*dt*)_max⁡_ was only slightly changed (from 1780 mm Hg/s in control to 1740 mm Hg/s at constant EMD). An analysis of wall tension development during IVC under constant EMD revealed that this effect arose from a delay in the onset of contraction in the midmyocardial and subepicardial layers (Figure 3(b)) causing initial deceleration of the pressure rise.

To reveal the physiological effects of transmural differences in MSV on development of intraventricular pressure during IVC, we performed further simulation under condition of constant MSV in all ventricular layers. The results illustrated by the dotted line in Figure 3(a) show that this change would lead to a decreased *dp*/*dt* during the whole IVC. This would cause a reduction of (*dp*/*dt*)_max⁡_ from 1780 mm Hg/s (control) to 1110 mm Hg/s and a prolongation of IVC from 60 ms (control) to 94 ms. Both changes are consistent with a decreased contractility and performance of LV. A change of tension development in individual ventricular layers underlying this effect is illustrated in Figure 3(c). As shown in the figure the rise of tension in individual layers is slowed down, which is the reason of the decreased dp/dt during IVC under this condition.

A combined effect of constant EMD and MSV on the time course of intraventricular pressure rise during IVC is shown by the dashed-dotted line in Figure 3(a). As apparent from the figure the model predicts that these conditions would cause a similar reduction of (*dp*/*dt*)_max⁡_ as in the case of constant MSV only (from 1780 mm Hg/s (control) to 1100 mm Hg/s). However, the prolongation of IVC duration would be more pronounced (from 60 to 104 ms). Hence the model shows that (*dp*/*dt*)_max⁡_ is almost exclusively affected by changes of MSV while the IVC duration is sensitive to changes of both MSV and EMD. These results suggest that while the homogenisation of both MSV and EMD across the LV wall contributes to a decreased contractility of LV, a reduction of transmural gradient of MSV plays a major role in this effect (see Table 1 for comparison).

## 4. Discussion

We present a new FE model of LV designed to simulate the effects of transmural differences in electromechanical characteristics of ventricular myocytes on development of tension in LV wall and intraventricular pressure during IVC. Here, a special attention is paid to exploration of the physiological role of transmural differences in EMD and MSV in these events.

### 4.1. Electrophysiological Contexts of Transmural Differences in Electromechanical Delay and Myocyte Shortening Velocity in Left Ventricle

The heterogeneity in electrophysiological properties of myocytes across the LV wall is commonly known. Substantial transmural distinctions in characteristics of membrane currents and action potential configurations have been demonstrated in myocytes from various species including humans [18, 35–41]. Related transmural distinctions in the excitation-contraction coupling have been also documented [35, 42–44]. However, transmural differences in mechanical activity of cardiomyocytes are still not conclusively explained and available experimental evidence is not fully consistent. In 2004, Cordeiro et al. [17] published their unique study examining the transmural heterogeneity of ionic currents, Ca^2+^ handling, and mechanical function in canine left ventricle. They identified several key distinctions in the time course of unloaded shortening among subepi-, midmyo-, and subendocardial cells. Subendocardial cells displayed a substantially longer EMD, while subepicardial cells showed the fastest MSV. Faster shortening of subepicardial cells than of those in the other myocardial layers was also observed in guinea pig hearts [45, 46] and in porcine multicellular preparations [47]. According to the coupled model of myocyte electromechanics designed by Campbell et al. [48], the distinctions in EMD in different cell types result from differences in the slope of the rising phase of intracellular Ca^2+^ transient. This interpretation is consistent with the observed transmural differences in a delay between onset of action potential (AP) and Ca^2+^ transient in human hearts [43]. As for the faster kinetics of subepicardial cells shortening, this phenomenon has been explained by the following: (i) higher activation of L-type calcium current (*I*_Ca_) during the early transient phase of AP repolarization induced by the presence of larger transient outward potassium current (*I*_Kto_) [17, 30, 48]; (ii) higher content of Ca^2+^ in the sarcoplasmic reticulum (SR) induced by the increased expression of SR Ca^2+^-ATPase [43, 44, 49]; and (iii) elevated expression of *α* myosin heavy chain (MHC) isoform in subepicardial myocytes which exhibit faster cross-bridge cycling kinetics [47, 50]. Besides, transmural differences in stiffness of cytoskeleton proteins [51] and in density of t-tubules as found in right atrium [52] might also play a potential role. Both shorter EMD and higher MSV in subepicardial myocytes are considered to compensate for the delayed arrival of AP into subepicardial layers, allowing synchronous contraction across the LV wall [17, 30]. This notion is highly consistent with numerous experimental and modelling studies showing that transmural fibre stress and strain are rather uniform in both diastole and systole (for review see Carruth et al. [53]). However, in vivo measurements of electrical activity and transmural myofibre mechanics in adult mongrel dogs during normal sinus rhythm or atrial pacing showed a surprisingly significant transmural gradient in the onset of myofibre shortening; the mean propagation velocity of myofibre shortening from endocardium to epicardium was 0.25 m/s [54]. In addition, the recent study on preparations from nonfailing human hearts indicated that shortening velocity of myocytes from different regions of LV does not exhibit significant differences [55]. Reasons of these contradictions are unclear. This would mean that a great part of energy produced in the heart is expended for mutual mechanical interaction between active and nonactive parts of the tissue which would decrease the pump efficiency of the heart.

### 4.2. Physiological Importance of Transmural Differences in Electromechanical Delay and Myocyte Shortening Velocity in Human Left Ventricle

**Figure 4:**
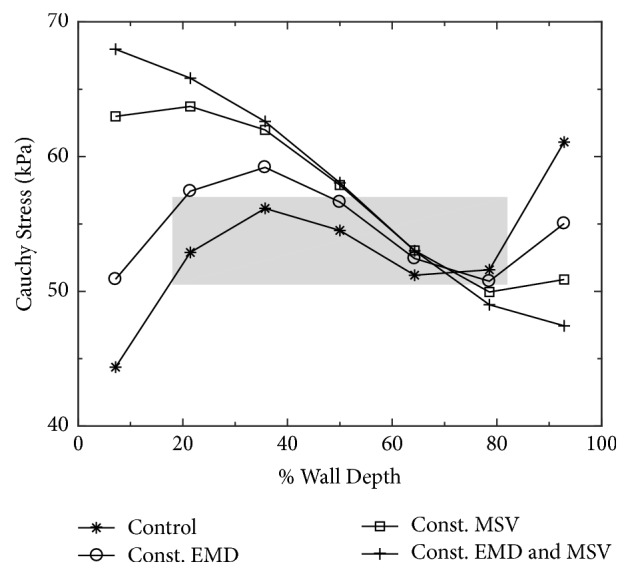
Change of stress along the direction of myofibres across the LV wall (from endocardium: 0% to epicardium: 100%) in the end of IVC, under control conditions and when either EMD, or MSV, or both of them were set constant in all ventricular layers. The shaded area shows that the transmural change of stress in the inner parts of LV is reduced in control conditions.

Optimal function of the heart depends on ordered mechanical events that are orchestrated by timing of electrical events. This electromechanical coupling occurs at multiple anatomic levels: within atria, between atria and ventricles, between ventricles, and especially within LV. It was postulated that transmural uniformity of systolic fibre stress and strain likely helps to maximize the efficiency of conversion of regional contractile work into pumping function of the heart [56]. Synchronous onset of contraction in all model layers (see Section 2.2) resulting from the combination of transmural change of EMD (set on the basis of measurements done by Cordeiro et al. [17]) and average conduction velocity of 47 cm/s [25, 26] supports this view. The model simulations also showed that if one or both of these cellular parameters were set constant across the LV wall, the nonuniformity in fibre stress in inner parts of the wall increased remarkably (see the changes of Cauchy stress between 20 and 80% of wall depth in Figure 4). This was associated with a prolonged duration of IVC (by 18 to 73%) and reduced (*dp*/*dt*)_max_ (by 2 to 38%, see Figure 3(a) and values in Table 1), indicating that a transmural homogenisation of electromechanical properties of ventricular myocytes impairs the function of the LV. Hence, our computations suggest that the transmural differences in the latency to onset of contraction and in the contraction velocity of myocytes play a physiologically important role in coordination of contraction of individual layers of ventricular myocardium, thereby ensuring a normal and most efficient LV function.

### 4.3. Clinical Implications

The results of our computational study indicate that a reduction of transmural gradients of EMD and MSV decreases the contractile function of LV because of discoordination of contraction in individual layers of LV. Such intraventricular dyssynchrony would lead to a decline in systolic performance, decreased mechanical efficiency, and a greater metabolic cost of LV contraction. Some changes initiated at cellular level can be identified to contribute to this pathological state. It has been documented that, in heart failure (HF), a greater reduction of *I*_Kto_ in subepicardial myocytes diminishes the LV transmural gradient of early repolarization [57]. Given the relation among early repolarization, magnitude of *I*_Ca_, rate of rise of intracellular Ca^2+^ transient, and dynamics of cellular contraction [19, 58, 59] it is obvious that a reduction of subepicardial *I*_Kto_ is an important factor underlying the attenuation of transmural gradient of EMD and MSV. It also appears that downregulation of *α* MHC isoform [60, 61] may contribute to the transmural reduction of both of these gradients because it exhibits higher expression in subepicardial myocytes [47]. Besides, transmural differences in disruption of excitation-contraction coupling induced by remodelling of t-tubules [62] may also play a role in this effect. On the other hand, an unphysiological increase of transmural gradients of EMD and MSV would lead to transmural desynchronization of contraction as well. It may happen for example under higher upregulation of Na-Ca exchanger current (*I*_NaCa_) or under higher downregulation of SR Ca^2+^ pump (SERCA) in subendocardial and midmyocardial cells compared to subepicardial cells as observed in HF [44, 63, 64]. Hence, all the above pathological changes typical for impaired function of the LV reduce its contractility in two ways: primarily because they decrease the contractile power of myocytes and secondarily because these changes in myocytes contractile power induce their nonsynchronous contraction across the LV wall. This complex effect of cellular impairment on LV function calls for therapeutic strategies that would not only restore the physiological concentrations of Ca^2+^ in cardiomyocytes (e.g., by inhibition of sodium-potassium pump or by stimulation or overexpression of SERCA) but also transmurally resynchronise their contraction. A supplementary pharmacological stimulation of subepicardial *I*_Kto_ may be one of the promising ways to achieve this aim.

### 4.4. Limitations of the Model

The FE model used in this study succumbs to several limitations. Firstly, the model includes only the LV with its geometry approximated by two truncated ellipsoids. The omission of the mechanical interaction between the ventricles, together with the simplification of ventricular shape, affects the motion of the ventricle due to passive and active forces generated within myocardium and thus may slightly influence the calculated results. The kinematics of the ventricle is also affected by the simplified boundary condition at the base which prevents the basal movement of the ventricle. On the other hand, the method of reconstruction of the unloaded geometry is fully applicable to patient-specific geometries recorded under end-diastolic intraventricular pressure. Secondly, the activation pattern used in the model assumes simultaneous depolarization of the whole subendocardial layer and the subsequent spread of excitation towards the epicardium. This is a simplification of the real activation sequence which starts from a few early activated areas in subendocardium and proceeds around the cavity and towards the epicardium [65]. However, since the spread around the cavity is much faster than that in the transmural direction [65], the simultaneous activation of subendocardial layer represents a reasonable approximation. Thirdly, the mechanical response of passive myocardium is orthotropic rather than transversely isotropic because of the myocyte sheet structure [23, 66]; although the myocardium anisotropy is dominated by myofibre directions, a fully orthotropic hyperelastic model could provide even more accurate constitutive description of passive myocardium. Finally, the time course of contraction was derived on the basis of the assumption of linear shortening of unloaded myocytes. We believe that these limitations may change the results quantitatively but without a significant impact on the drawn conclusions.

### 4.5. Applicability of Model Results to Other Species

The results presented in this study hold true for humans and, to certain extent, for species with similar LV geometry, electromechanical properties of cardiomyocytes, and their similar interconnection with ventricular conduction system (Purkinje fibres). While it is clear that the changes in LV size and shape at the preserved transmural pattern of contraction would lead to substantial changes in the development of intraventricular pressure the impact of the two other factors is not clearly apparent. Nevertheless, considering the potential role of transmural gradient of *I*_Kto_ in synchronisation of contraction across the human LV wall (see Section 4.2) it seems to be obvious that different mechanisms play the same role in other species that lack *I*_Kto_ (e.g., guinea pig [67]) or have a slowly recovering *I*_Kto_ (e.g., rabbit [68]). To unhide these mechanisms, special models accounting for these interspecies differences would be needed. Regarding the interconnection between conduction system and working myocytes, our model considered that the Purkinje fibres contact the endocardial surface at discrete points (resulting in near simultaneous activation) as has been found in human hearts [69]. However, beside endocardial also intramural Purkinje fibres have been demonstrated in ungulates including sheep [70], cow [71], pig [69], and horse [72], as well as in whales [73]. This implies that, unlike in our model, both endo- and midmyocardium are activated at the same time in these species. A tentative incorporation of this finding into our model led to transmural desynchronization of contraction starting in epicardial layers and propagating towards endocardium. The effect of this intervention on LV contractility was similar to that of constant EMD in our simulations (see Figure 3(a)) but less pronounced (IVC increased to 65 ms only and (*dp*/*dt*)_max⁡_ remained unchanged).

## 5. Conclusion

The presented results suggest that transmural differences in EMD and MSV play an important role in optimization of contractile function of LV. The control model with EMD and MSV distribution prescribed on the basis of experimental results showed the highest contraction efficiency and the increase of intraventricular pressure corresponding to physiological curves. In cases with the values of EMD, MSV, or both kept constant across the LV wall the IVC was prolonged and (*dp*/*dt*)_max⁡_ was reduced. This reduction of contractile power was accompanied by remarkable changes in distribution of LV wall stress. As the contractile properties of living myocytes are governed to a large extent by transmembrane ionic currents, their differential region-dependent pharmacological modulation (e.g., selective activation of *I*_Kto_ in subepicardial myocytes) might be beneficial in clinical treatment of HF.

## Figures and Tables

**Table 1:** Values of (*dp*/*dt*)_max⁡_ and of IVC duration obtained from the model simulations under specified conditions.

	(*dp*/*dt*)_max⁡_ [mm Hg/s]	IVC [ms]
Control	1780	60
Constant EMD	1740	71
Constant MSV	1110	94
Constant EMD and MSV	1100	104
